# Daily Amount of Purine in Commonly Recommended Well-Balanced Diets in Japan and Overseas

**DOI:** 10.3390/nu16234066

**Published:** 2024-11-27

**Authors:** Kiyoko Kaneko, Keito Tsuruga, Fukue Takayanagi, Tomoko Fukuuchi, Noriko Yamaoka, Reiko Seki, Shin Fujimori

**Affiliations:** 1 Biomolecular Logic Research Laboratory, Kita-ku, Tokyo 114-0023, Japan; 2 Faculty of Pharmaceutical Sciences, Teikyo University, Itabashi-ku, Tokyo 173-8605, Japan; 3 FORALL Co., Ltd., Koutou-ku, Tokyo 136-0072, Japan; 4 Urayasu Takayanagi Hospital, Urayasu-shi, Chiba 279-0004, Japan; 5 Corporation Tsubasa Ryougoku East Gate Clinic, Sumida-ku, Tokyo 130-0026, Japan

**Keywords:** amount of purine, gout and hyperuricemia, well-balanced diet, food guidelines, Mediterranean diet, DASH diet

## Abstract

**Background/Objectives:** A low purine diet has been recommended for patients with gout and hyperuricemia, but there are concerns about excessive carbohydrates and a lack of protein. A well-balanced diet in accordance with general dietary guidelines is widely recommended. The Mediterranean diet and the DASH (Dietary Approaches to Stop Hypertension) diet are also recommended for gout and hyperuricemia patients. However, there is little information on the purine levels in these diets. The aim of this study is to determine the daily amount of purine in well-balanced diets that follows the dietary guidelines, including the Mediterranean diet and the DASH diet. **Methods**: We measured the purine content in various foodstuffs. Using these values, we calculated the amount of purine in generally recommended well-balanced diets—the Japanese diet, American My Plate Plan, the Mediterranean diet, and the DASH diet. To calculate the amount of purine, recipes tailored to the characteristics of each diet were created. **Results**: The amount of purine in the Japanese diet, in the American My Plate Plan, in the Mediterranean diet, and in the DASH diet were 308.5–366.1, 308.7–335.0, 346.6–394.1, 325.9–493.9 mg/day, respectively. These values were close to the 400 mg/day recommended in the Japanese Guideline for the management of Hyperuricemia and Gout. **Conclusions**: A well-balanced diet following the recommendation in the dietary guidelines is considered to provide adequate purines. Because a high purine diet can lead to the recurrence of gout, advising to eat a balanced diet is useful on lifestyle guidance for the patients with gout and hyperuricemia.

## 1. Introduction

**Figure 1:**
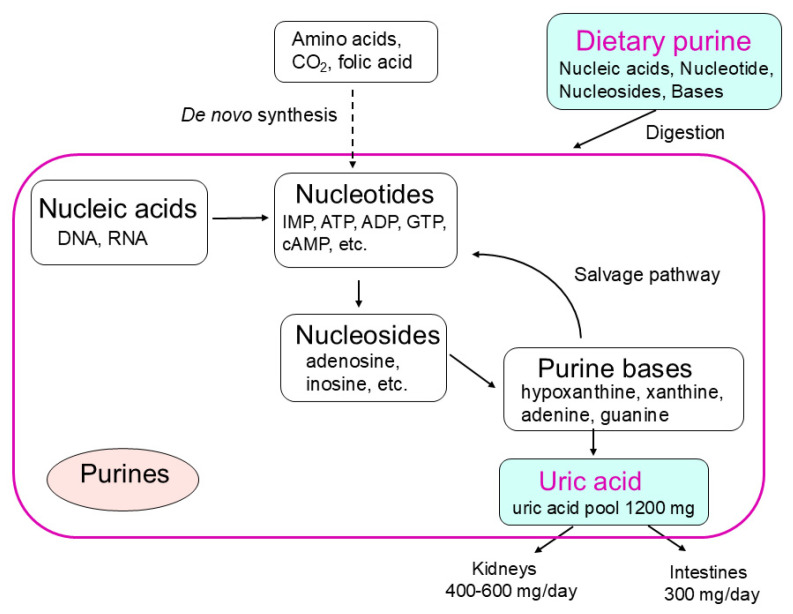
Purine metabolism and Dietary purine.

Purines that have a purine ring as a chemical structure contain various types including nucleic acids such as DNA and RNA, nucleotides such as ATP and GMP, nucleosides such as adenosine, and purine bases. These purines in the body are used for cell division, energy metabolism, and signal transduction, and in humans, are ultimately excreted from the kidneys and intestinal tract as uric acid (Figure 1). Uric acid is mainly produced in the liver, circulates in the blood, and about two-thirds is excreted in urine from the kidneys and about one-third is excreted in feces from the digestive tract [1]. Since uric acid is poorly soluble in water, excess uric acid not only increases serum uric acid levels but also crystallizes as monosodium urate crystals in joints and other areas, causing gouty arthritis.

Foods and beverages that we consume also contain cellular nucleic acids, nucleotides, nucleosides, and bases that are converted into uric acid. Purines are a source of energy for the body, especially for the intestinal tract, but excessive intake of purine can increase serum uric acid levels. It has been known since medieval Europe that gout was common among gourmets and heavy drinkers, and that there is a link between diet and gout [2]. A low purine diet has been recommended for patients with gout and hyperuricemia, but there are concerns about excessive carbohydrates and a lack of protein [3].

The number of patients with gout is rising in developed countries, not only in Japan. According to the Basic Survey on National Life in Japan, the number of patients who visited hospitals for gout (gouty arthritis) in 2022 was 1.236 million men and 70,000 women, totaling 1.306 million, which, for both genders, shows increments from 2019 (1.195 million men, 59,000 women, totaling 1.254 million) and 2016 (1.048 million men, 57,000 women, totaling 1.105 million). Relative to gout patients since 1998, the number has increased by about 100,000 every three years on average [4].

Hyperuricemia is defined as a serum uric acid level above 7.0 mg/dL in both men and women [5]. Hyperuricemia causes uric acid deposition diseases (gouty arthritis, renal disorders, etc.) [6], and the number of patients is estimated to be about 10 times that of gout patients, so the number of hyperuricemia patients in Japan is thought to exceed 13 million. The frequency of hyperuricemia is one in four or five adult men, which is the same as in other countries [7].

It has been reported that hyperuricemia is associated with increased BMI and hypertension [8], and has been listed as a risk factor of cardiovascular disease that affects life prognosis [9]. Hyperuricemia is also associated with renal disorders, urinary stones, diabetes, and metabolic syndrome. It is important to keep serum uric acid levels within the standard range in order to prevent other lifestyle-related diseases [5]. In particular, ischemic cardiovascular diseases such as strokes have a high prognosis of being bedridden, so preventing hyperuricemia is important in terms of extending healthy life expectancy [9].

A report examining the relationship between diet and serum uric acid levels also found that the more meat and fish consumed, the higher the serum uric acid level and the higher the risk of developing gout [10]. It has been reported that excessive intake of purines increases the risk of gout recurrence [11]. According to a meta-analysis summarizing foods and the risk of hyperuricemia, foods that increase serum uric acid level include red meat, seafood, alcohol, and fructose [12]. Thus, the 2020 ACR guideline for the management of gout conditionally recommends limiting purine intake [13].

A study addressing the possibility of dietary prevention on serum urate and cardiovascular disease found that significant benefits were seen following a low-fat, Mediterranean, or low-carbohydrate weight loss diets [14]. The Mediterranean diet/DASH (Dietary Approaches to Stop Hypertension) has been reported to lower serum uric acid levels [15,16] and reduce cardiovascular risk factors in other at-risk populations [17]. Diet plays an important role for patients with gout and hyperuricemia [16,18].

Although there are reports on the purine content of foods [19], there are few reports on the amount of purine in the diet [20,21], and, to our knowledge, no reports on the Mediterranean diet or DASH diet. A balanced diet in accordance with general dietary guidelines is also recommended for patients with gout and hyperuricemia. Thus, in this study, we compiled the purine content of diets that are considered to be effective for patients with gout and hyperuricemia, specifically, generally recommended well-balanced diets based on the dietary guidelines, the Mediterranean diet, and the DASH diet.

## 2. Methods

### 2.1. Determination of Purine Content in Food

We continued to measure the purine content of foods and improved our methods [19,22]. The foods were purchased from retail stores, pretreated, hydrolyzed, and analyzed by high-performance liquid chromatography (HPLC). Briefly, food samples were hydrolyzed with 70% perchloric acid at 95 °C under stirring for 60 min. By way of this process, the nucleic acids, nucleotides, and nucleosides present in the samples were converted to the corresponding bases: adenine, guanine, hypoxanthine, and xanthine. These four purine bases were then accurately identified and quantified by HPLC implementing the peak-shift method. After adding xanthine oxidase (8.9 U/mL) and glycylglycine buffer (pH 8.2) to obtain the reaction solution of the sample, mixture was incubated at 37 °C for 3 h and was subjected to our inspection of the disappearance of the peaks of all analytes. Total purine content was calculated by the sum of each purine base

HPLC conditions were as follows: instrument, Shimadzu LC10A HPLC system with autoinjector SIL-10AD (Kyoto, Japan); column, Shodex Asahipak GS-320HQ (7.6 mm i.d. and 300 mm length) (Tokyo, Japan); mobile phase, 150 mM sodium phosphate buffer (pH 2.5–2.8); flow rate, 0.6 mL/min; column temperature, 35 °C; and detector wavelength, 260 nm.

### 2.2. Typical Dietary Guideline Survey

**Figure 2:**
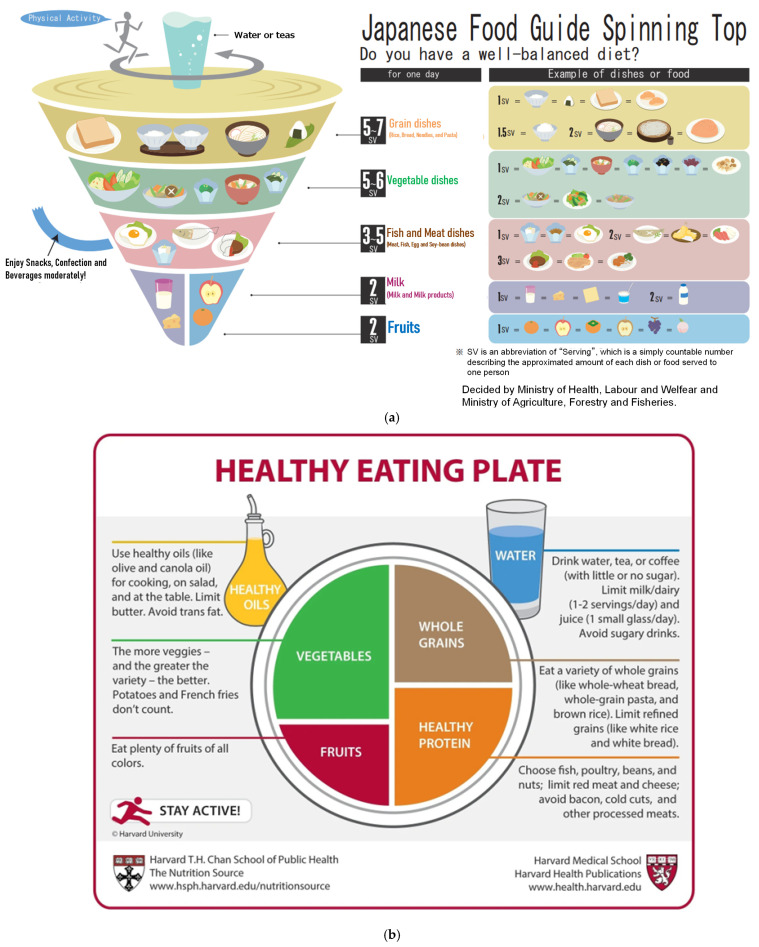

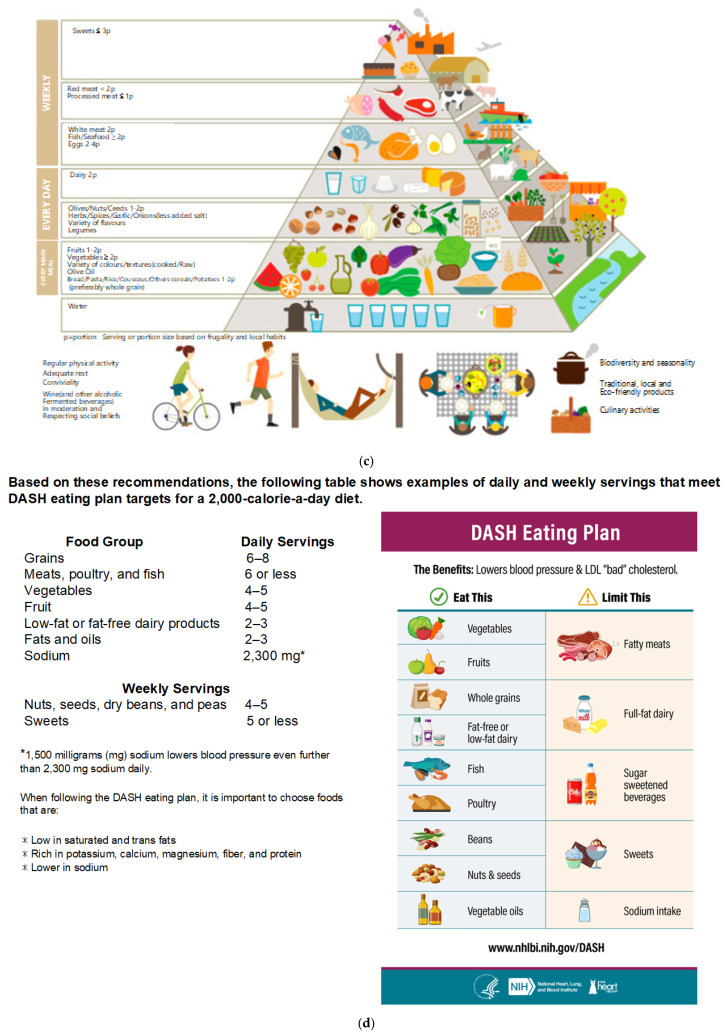
(**a**) Japanese Food Guide (Japanese Food Guide Spinning Top). The Ministry of Agriculture Forestry and Fishers. The Ministry of Health Labor and Welfare Japanese Food Guide Spinning Top. Available online: https://www.fao.org/nutrition/education-nutritionnelle/food-dietary-guidelines/regions/japan/en/ (accessed on 21 November 2024). (**b**) American Healthy Eating Plate (My Plate Plan). U.S. Department of Agriculture. MyPlate: Available online: https://www.myplate.gov/eat-healthy/what-is-myplate (accessed on 21 November 2024). (**c**) Mediterranean diet [27]. Serra-Majem, L. et al. (**d**) DASH diet. U.S. National Heart, Lung, and Blood Institute: Available online: https://www.nhlbi.nih.gov/education/dash-eating-plan (accessed on 21 November 2024).

As typical dietary guidelines, we investigated the Dietary Reference Intake and Dietary Guideline [23,24]. For Japanese patients, we referred to the Japanese Food Guide Spinning Top that is designed by the Ministry of Agriculture, Forestry, and Fisheries of Japan (Figure 2a) [25]. For patients in USA, the My Plate Plan [26] proposed by the U.S. Food and Drug Administration (FDA) and United States Department of Agriculture (USDA) were referred to (Figure 2b). As both the Mediterranean diet [27,28] and the DASH diet [29,30] were reported to be effective for patients with gout or hyperuricemia through several studies [3,14,15,16,17,18], diets based on these eating patterns were also used as a guide (Figure 2c,d).

### 2.3. Creating Recipes for Each Dietary Guideline and Calculating Daily Purine Amount

Recipes were created based on the Japanese Food Guide Spinning Top and the Japan Diet [31], the My Plate Plan, the Mediterranean diet, and the DASH diet [23,24,27]. These dietary guidelines have their own recommended ingredients and quantities, as well as frequency of intake, as shown in Figure 2. We created three days’ worth of recipes based on the characteristic instructions, then we calculated the daily purine amount in each diet. Recipes for the Mediterranean diet and the DASH diet were created by combining recipes from books that described each diet and menu, to fit the instructions in the guidelines [32,33]. When calculating daily purine amount, we used our reported data and, if the purine content of a food was not measured, the value of a similar food was used instead of that.

The Japan diet is a dietary pattern derived from *washoku* (Japanese cuisine) that has been registered in 2013 as a world intangible cultural heritage by United Nations Educational, Scientific and Cultural Organization (UNESCO), and is a dietary approach recommended by the Japan Atherosclerosis Society [31,34]. In Japan, the basic meal combination of a staple food, main dish, one or two side dishes, and soup has long been advocated, as recommended in the Japan Diet. The Japan Diet is a dietary method targeted at patients with high risk of arteriosclerosis, who have high levels of LDL-cholesterol or triglycerides. For this reason, eggs are listed as a food to be avoided. In contrast, our study targeted patients with hyperuricemia and gout. Therefore, a recipe was created using eggs, which contain almost no purines that are the precursor of uric acid, and which are a good source of protein.

**Figure 3:**
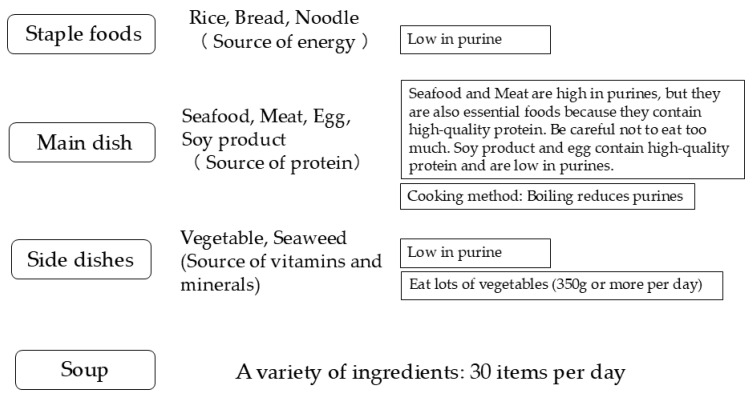
Japanese dietary pattern with some key points regarding purine.

Figure 3 summarizes traditional Japanese dietary pattern, as typified by *washoku* and the Japan Diet, along with some key points to consider regarding purines. A typical combination of Japanese diet includes a staple food, a main dish, one or two side dishes, and soup. Seafood and meat are high in purines whereas other foods (grains, soy product, vegetable, egg, seaweed, mushroom) are low in purines. It is also recommended to eat a balanced diet containing an appropriate amount of energy according to age, height and physical activity, aiming for 30 different types of food per day.

## 3. Results

### 3.1. Purine Content of Commonly Used Foodstuffs

The purine content of commonly used foodstuffs is shown in Table 1. In addition to previous report [19], recently measured and unpublished purine contents are also shown [35,36]. Purine content is shown per 100 g by the food. Dry foods have a lower moisture content and condensed purines, so they show higher values.

Purines are generally found in plants and animals, and the parts of them that have a large number of cells, or where cell division is active, contain a high amount of purine. Purine content is high in meat, fish, and seafood derived from animals, and low in vegetables and fruits derived from plants.

Purines are mainly derived from nucleic acids, nucleotides, and nucleosides, and are therefore water-soluble. Accordingly, the purine content is reduced by boiling and is lower in canned and processed foods than in the original ingredients.

### 3.2. Japanese Well-Balanced Diet and a Daily Amount of Purine in It

**Figure 4:**
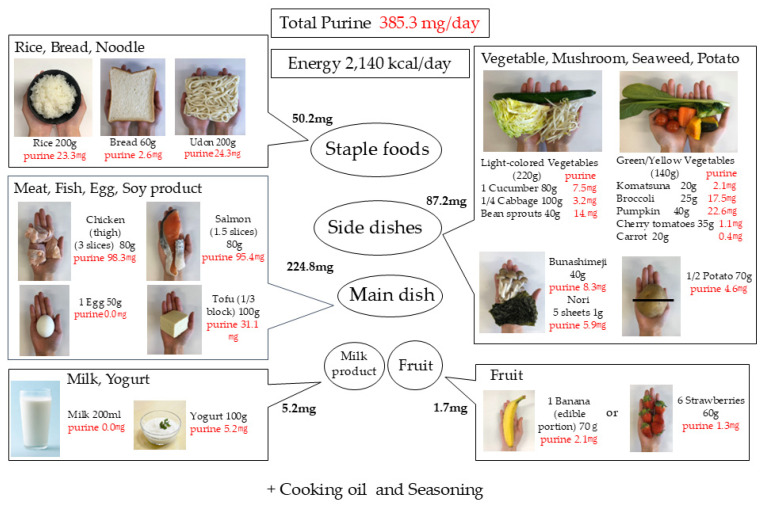
A well-balanced diet based on the Japanese Food Guide Spinning Top.

A well-balanced diet was determined by aligning ingredients and portions with the Japanese Food Guide Spinning Top and by consuming foods in accordance with traditional Japanese eating habits (Figure 4). This diagram was created to help users visualize the recommended daily amount of intake when they hold each ingredient in their hand.

Staple foods were divided into three meals and consisted of rice, bread, and udon noodles, with purine contents of 23.3 mg, 2.6 mg, and 24.3 mg, respectively. It is recommended that the ratio of light-colored vegetables to colored vegetables be 2:1 for side dishes. This is 220 g of light-colored vegetables and 140 g of colored vegetables, for a total of 360 g, which meets the daily vegetable intake of 350 g or more recommended by Health Japan 21 [37]. For the main dish, it is recommended to have both meat and fish every day. Therefore, we chose chicken and salmon from the frequently eaten foods, along with tofu as a soy product, and eggs. Eggs are often mistakenly thought to contain a lot of purines, but the purine content was almost 0 mg/100 g. The amount of purine from the main dish and side dishes was 224.8 mg and 87.2 mg, respectively. It is recommended to eat one type of fruit per day, but care must be taken not to consume too much fructose in fruit. It has also been reported that the intake of milk and dairy products reduces serum uric acid levels, so it is recommended to actively consume them. The amount of purine from fruits and dairy products was 1.7 mg and 5.2 mg, respectively.

The total daily amount of purine and the daily energy derived from these food ingredients were 369.1 mg and 1930 kcal, respectively. Seasonings and cooking oil are used in cooking, so we add the amounts (purine: 16.2 mg, energy: 210 kcal) calculated from Table 2, the total comes to 385.3 mg and 2.140 kcal, respectively. This amount of purine is in line with the recommended daily purine intake of about 400 mg as set out in the Japanese Guideline for the Treatment of Hyperuricemia and Gout [5].

### 3.3. Recipes Based on Dietary Guidelines and Daily Amount of Purine

The recipes created in accordance with each guideline, along with their respective energy intake and purine amounts, are shown in Table 2, Table 3, Table 4 and Table 5.

The Japanese diet shown in Table 2 consists of three meals—breakfast, lunch and dinner—and uses grains, meat, fish, vegetables, soy product, seaweed, dairy products, and fruit. These recipes are generally considered to feature well-balanced meals that include multiple dishes, such as a main dish, side dishes, staple foods, and a soup. The daily amount of purine and energy intake of these recipes are 308.5–366.1 mg and 1830–1918 kcal, respectively.

The recipes created based on the American Healthy Eating Plate, the My Plate Plan, are shown in Table 3. It consists of three meals—breakfast, lunch and dinner—and uses unrefined grains, beans, fish or seafood, meat, vegetables, dairy products, soy product, and fruit. The recipes are designed to help us consume the recommended amounts of fruits, vegetables, grains, protein, and dairy products using My Plate Plan, resulting in a balanced diet. The daily amount of purine and energy intake of these recipes are 308.7–335.0 mg and 1832–2010 kcal, respectively.

The recipes created according to the Mediterranean diet are shown in Table 4. It consists of four meals—breakfast, snacks, lunch, and dinner—and uses vegetables, beans, nuts, unrefined grains, fish or seafood, meat, dairy products, and fruit. The Mediterranean diet recommends lunch as the main meal, and that we consume a lot of whole grains, seasonal vegetables and fruits, legumes, nuts, and fish and seafood. These recipes are created in accordance with this characteristic and are considered to achieve a balanced meal. The daily amount of purine and energy intake based on these recipes are 346.6–394.0 mg and 1510–2287 kcal, respectively.

In Table 5, the recipes created according to the DASH diet are indicated. It consists of five meals—breakfast, snacks, lunch, snacks, and dinner—and uses vegetables, legumes/beans, mushrooms, fish or seafood, meat, dairy products, and fruit. The DASH diet recommends a focus on whole grains, vegetables, and fruits, along with fish, poultry, beans, nuts, and low-fat dairy products. These recipes are designed to follow this principle to provide a balanced diet. The daily amount of purine and energy intake of these recipes are 325.9–493.9 mg and 1600–1713 kcal, respectively. Since 200 g of chicken (which contains more purine than other meats (Table 1)) is consumed at dinner in recipe 2, the daily amount of purine is increased. But the energy intake of the meal was as low as 265 kcal.

**Table 2:** Japanese diet based on the Japanese Food Guide Spinning Top.

Recipe 1				Recipe 2				Recipe 3		
Breakfast	Energy (kcal)	Purine (mg)		Breakfast	Energy (kcal)	Purine (mg)		Breakfast	Energy (kcal)	Purine (mg)
Bread	196	2.6		Bread	196	2.6		Bread	196	2.6
(Bread, Orange Marmalade)				(Bread, Strawberry jam)				(Bread, Orange Marmalade)		
Egg Salad	138	2.8		Egg soup	124	2.7		Boiled Egg	76	0
(Lettuce, Cucumber, Onion, Egg, Sesame dressing)				(Whole eggs, Celery, Carrots, Onions, Consomme, Sesame oil, Starch)				(Egg, Salt)		
Fruit	38	1.3		Fruit	86	3.0		Spaghetti Salad	132	2.4
(Pineapple)				(Banana)				(Spaghetti, Cabbage, Cucumber, Onion, Carrot, Mayonnaise)		
Milk	121	0.3		Milk	121	0.3		Fruit	86	3.0
(Milk)				(Milk)				(Banana)		
Breakfast subtotal	493	7.0		Breakfast subtotal	527	8.6		Milk	121	0.3
								(Milk)		
Lunch				Lunch				Breakfast subtotal	611	8.3
Rice	322	23.3		Rice	322	23.3				
(Rice (polished))				(Rice (polished))				Lunch		
Swordfish with Ginger and Soy Sauce	232	114.3		Deep-fried Chicken with Green Beans	189	102.7		Kenchin udon	408	96.0
(Swordfish, Wheat flour, Salad oil, Mirin, Ginger, Reduced-sodium soy sauce, Broad beans)				(Chicken thighs, Ginger, Cooking sake, Starch, Frying oil, Green beans, Reduced-sodium soy sauce)				(Udon, White Radish, Carrots, Chicken thighs, Fried Tofu, Konnyaku, Burdock, Soup stock, Mirin, Reduced-sodium soy sauce, Snow Peas)		
Ratatouille	63	17.5		Potato Kinpira	93	6.4		Noodle Sauce, Shichimi Pepper	29	21.4
(Boiled Soybeans, Vegetable mix, Onion, Tomato, Garlic, Olive oil, Consomme)				(Potatoes, Red Peppers, Salad oil, Sugar, Reduced-sodium soy sauce, Sesame oil)				(Noodle Soup, Dashi stock, Bonito powder, Shishito Pepper)		
Miso soup	28	22.9		Two-color dipping	14	21.5		Tossed with ponzu sauce	68	30.2
(White Miso, Bean sprouts, Dashi stock)				(Spinach, Chinese Cabbage, Reduced-sodium soy sauce, Dashi stock, Bonito flakes)				(Chinese Cabbage, Mitsuba, Canned Tuna, Ponzu Sauce)		
Fruit	31	1.6		Lunch subtotal	618	153.9		Shiratama Zenzai	178	17.9
(Apple)								(Shiratama Dumplings, Red Beans, Sugar, Salt)		
Lunch subtotal	676	179.6						Lunch subtotal	683	165.5
Dinner				Dinner				Dinner		
Rice	322	23.3		Rice	322	23.3		Rice	322	23.3
(Rice (polished))				(Rice (polished))				(Rice (polished))		
Tofu and Beef	224	90.2		Beef Curry	283	95.3		Salmon Meuniere with Potato & Lemon	186	148.5
(Grilled tofu, Beef thigh, Onion, Parsley, Dashi stock, Cooking sake, Mirin, Reduced-sodium soy sauce, Chrysanthemum)				(Beef thigh, Garlic, Ginger, Onion, Carrot, Butter, Salt, Curry powder, Canned Tomatoes, Red Wine, Worcestershire sauce, Fukujinzuke)				(Salmon, White cooking Wine, Flour, Salad oil, Butter, Potatoes, Dried Parsley, Lemon)		
Pumpkin Salad	92	34.5		Pickles	27	26.4		Lotus Root Kinpira	99	12.3
(Pumpkin, Onion, Mayonnaise)				(Celery, Cucumber, Cauliflower, Red Pepper, Vinegar, Sugar)				(Lotus Root, Ground Beef, Sesame oil, Mirin, Sugar, Reduced-sodium soy sauce)		
Radish tossed in Yukari Sauce	23	8.6		Chinese dessert	78	1.0		Cucumber and Seaweed Vinegared Dish	17	8.1
(White Radish, Cucumber, Bamboo, Chikuwa, Yukari)				(Canned Chinese Dessert, Canned Fruit Cocktail, Canned Syrup)				(Mozuku Seaweed, Cucumber, Salt, Ginger, Sugar, Vinegar)		
Dinner subtotal	661	156.6		Dinner subtotal	710	146.0		Dinner subtotal	624	192.3
Daily total	1830	343.2		Daily total	1855	308.5		Daily total	1918	366.1

**Table 3:** My Plate Plan diet.

Recipe 1				Recipe 2				Recipe 3		
Breakfast	Energy (kcal)	Purine (mg)		Breakfast	Energy (kcal)	Purine (mg)		Breakfast	Energy (kcal)	Purine (mg)
Serial	198	18.2		Tomato Risotto with Oatmeal and Beans	231	62.3		Acai Bowl	450	101.7
(Corn flakes)				(Oatmeal, Kidney beans, Onions, Broccoli, Tomato juice, Low-fat milk, Consommé, Olive oil)				(Acai, Nonfat yogurt, Banana, Strawberry, Blueberry, Granola, Honey)		
Chickpea Salad	295	37.3		Frozen Yogurt	165	9.6		Egg Salad	183	6.5
(Cucumber, Lettuce, Cherry tomato, Onion, Sweet potato (steamed), Chickpeas, Olive oil, Lemon juice)				(Fat-free yogurt, Honey, Strawberries)				(Boiled egg, Cucumber, Lettuce, Cherry tomato, Olive oil, Salt, Black pepper)		
Yogurt	92	10.5		Breakfast subtotal	397	71.9		Breakfast subtotal	633	108.2
(Fat free yogurt, Raspberries, Blueberries)										
Breakfast subtotal	585	66.0								
Lunch				Lunch				Lunch		
Rye Sandwich	343	57		Bagel Sandwich	625	15.8		Tuna Sandwich	446	69.5
(Rye bread, Chicken (thigh, skinless, boiled), Leaf lettuce, Tomato, Cream cheese, Black pepper)				(Bagel, Fried egg, Avocado, Lettuce, Mayonnaise, Pepper)				(Bread, Tuna, Onion, Lettuce, Tomato, Mayonnaise, Pepper)		
Fruit	148	4.6		Clam Chowder	249.6	87.7		Minestrone	105	13.5
(Apple, Pineapple, Grapes)				(Clams (boiled), Bacon, Onion, Potato, Carrot, Flour, Low-fat milk, Salted butter, Pepper, Parsley)				(Carrots, Onions, Potatoes, Tomatoes, Cabbage, Canned green peas, Macaroni, Consommé, Parsley)		
Cafe Latte	72	0.2		Lunch subtotal	875	103.5		Soy Latte	74	30.1
(Coffee, Low-fat milk)								(Coffee, Soy milk)		
Lunch subtotal	563	61.9						Lunch subtotal	625	113.1
Dinner				Dinner				Dinner		
Seafood Doria	562	160.4		Bread	196	4.5		Garlic Toast	267	6.25
(Indica rice, Shrimp, Scallops, Squid, Onion, Spinach, Flour, Low-fat milk, Salted butter, Pepper, Breadcrumbs, Processed cheese, Parsley)				(Whole Wheat Bread)				(Whole wheat bread, Garlic, Butter, Parsley)		
Kale Salad	122	20.4		Salmon and Grilled Vegetables with Dill Yogurt Sauce	299	131.6		Pork and Beans	121	34.7
(Kale, Lettuce, Green pepper, Red pepper, Yellow pepper, Almonds (unsalted), Olive oil, Apple cider vinegar, Salt, Pepper)				(Salmon, Olive oil, Red pepper, Yellow pepper, Pumpkin, Dill, Nonfat yogurt, Salt, Pepper)				(Pork, Onion, Carrot, Pepper, Tomato puree, Garlic, Kidney beans, Salad oil, Chili powder, Consommé)		
Dinner subtotal	684	180.8		Fruit and Quinoa Salad	243	23.5		Avocado Salad	185	45.1
				(Lettuce, Celery, Quinoa, Orange, Grapefruit, Apple, Kiwi, Olive oil, Salt, Black pepper)				(Avocado, Chicken (Thigh, Skinless, Boiled), Lettuce, Tomato, Cottage Cheese, Cilantro, Lemon Juice, Salt, Pepper)		
				Dinner subtotal	738	159.6		Mango Smoothie	101	10.1
								(Mango, Nonfat yogurt)		
								Dinner subtotal	673	96.1
Daily total	1832	308.7		Daily total	2010	335.0		Daily total	1932	317.5

**Table 4:** Mediterranean diet.

Recipe 1				Recipe 2				Recipe 3		
Breakfast	Energy (kcal)	Purine (mg)		Breakfast	Energy (kcal)	Purine (mg)		Breakfast	Energy (kcal)	Purine (mg)
Almond Chia Porridge	150	15.7		Vegetable Breakfast Bowl	213	9.9		Mediterranean Breakfast Salad	121	56.7
(Almond milk, Chia seeds, Honey, Vanilla extract, Ground cardamom)				(Sweet potato, Russet potatoes, Onion, Paprika, Garlic powder, Onion poder, Olive oil, Sriracha sauce, Coconut milk)				(Egg, Arugula, Cherry tomato, Cucumber, Avocado, Quinoa, Chopped almond, Chopped herbs, Lemon, Olive oil, Salt, Pepper)		
Bread (Whole wheat bread)	226	5.2		Bread (Whole wheat bread)	113	2.6		Breakfast subtotal	121	56.7
Breakfast subtotal	376	20.9		Breakfast subtotal	326	12.5				
Snacks				Snacks				Snacks		
Cherry Tomato Bruschetta	100	8.5		Garlic Broccoli Rabe	87	69.3		Citrus Marinated Olives	200	6.4
(Cherry tomato, Chopped herbs, Whole grain bread, Ricotta cheese, Olive oil, Salt, Black pepper)				(Broccoli Rabe, Olive oil, Garlic, Red pepper flake, Salt, Black pepper)				(Green Olives, Garlic, Orange, Olive oil, Red wine vinegar, Red pepper flake, Ground cumin, Bay leaf)		
Lunch				Lunch				Lunch		
Cauliflower and Quinoa	220	72.7		Beans and Rice	224	38.3		Tomato and Millet Mix	222	17.3
(Quinoa, Cauliflower, Onion, Olive oil, Salt, Black pepper, Parsley)				(Rice unpolished, Canned Black Beans, Yellow onion, Celery, Garlic, Olive oil, Salt, Pepper, Water)				(Millet, Tomato, Onion, Olive oil, Coriander, Chili Paste, Lemon juice, Salt, Pepper)		
Mustard Chicken	531	89.9		Balsamic marinated pork loin skillet	309	112.3		Shrimp and Bean Salad	101	101.8
(Chicken breast, Chicken broth, Mustard, Olive oil, Paprika, Garlic powder, Chili Powder)				(Pork Tenderloin, Balsamic vinegar, Paprika, Honey, Onion, Zucchini, Garlic, Olive, Basil, Olive oil, Salt, Pepper)				(Shrimp, Kidney beans, Onion, Cherry tomato, Lemon peel, Olive oil, Salt, Pepper, Red wine vinegar)		
Roasted Brussels Sprouts with Orange	111	4.7		Sautéed Cabbage	117	6.2		Cauliflower with Kale Paste	41	31.6
(Brussels sprouts, Olive oil, Garlic, Orange, Salt)				(Cabbage, Onion, Olive oil, Parsley, Lemon Juice, Salt, Pepper)				(Cauliflower, Kale, Basil, Olive oil, Garlic, Lemon juice, Salt)		
Coconut Tahini Cashew Bars (No-Bake)	161	6.6		Roasted Fruit	60	9.2		Banana Ice Sundae	328	4.5
(Medjool dates, Walnuts, Cashew nuts, Coconut oil, Salt, Cashew Butter, Tahini)				(Peach, Blueberry, Cinnamon, Brown sugar)				(Banana, Almond milk, Cocoa powder, Hazelnut butter, Vanilla pod powder, Topping)		
Lunch subtotal	1023	173.9		Lunch subtotal	710	166.0		Lunch subtotal	692	155.3
Dinner				Dinner				Dinner		
Grilled Halibut	159	118.4		Fish with Tomato Sauce	86	86.0		Lemon-flavored Trout with Caramelized Shallots	344	154.3
(Halibut, Onion, Green pepper, Chicken stock, Olive oil, Cherry tomato)				(Codfish, Cherry tomato, Garlic, Chicken stock, Basil, Salt, Pepper)				(Trout(Salmon), Almond butter, Capers, Lemon juice, Lemon, Salt, Pepper, Almond butter, Onion)		
Vegetable with Avocado Dressing	403	47.8		Roasted Acorn Squash with Sage	188	10.4		Paprika and Chive Potato Chips	233	18.8
(Roasted pepitas, coriander, Parsley, Corn, White radish, Avocado, Greek Yogurt, Mango, Balsamic vinegar, Olive oil, Lime juice)				(Acorn squash, Sage, Thyme, Olive oil, No-salt Butter, Salt, Black pepper)				(Potato, Olive oil, Celery, Tomato, Paprika, Salt, Pepper, Chive)		
Bread (Whole wheat bread)	226	5.2		Bread (Whole wheat bread)	113	2.6		Bread (Whole wheat bread)	113	2.6
Dinner subtotal	788	171		Dinner subtotal	387	98.9		Dinner subtotal	690	175.6
Daily total	2287	374.7		Daily total	1510	346.6		Daily total	1703	394.0

**Table 5:** DASH diet.

Recipe 1				Recipe 2				Recipe 3		
Breakfast	Energy (kcal)	Purine (mg)		Breakfast	Energy (kcal)	Purine (mg)		Breakfast	Energy (kcal)	Purine (mg)
The Amazing Feta Hash	303	8.7		Peaches and Greens Smoothie	105	20.9		Sausage Casserole	74	36.3
(Hash browns, Low-fat feta (cheese), Eggs, Soy milk, Onion, Olive oil)				(Peaches, Spinach, Low-fat milk, low fat Greek yogurt, No-calorie seetener)				(Eggs, Onion, Sausage, Baby potatoes, Chili pepper, Olive oil)		
Bread (Whole wheat bread)	113	2.6		Bread (Rye bread)	151	3.4		Bread (Whole wheat bread)	226	5.2
Breakfast subtotal	416	11.3		Breakfast subtotal	256	24.3		Breakfast subtotal	300	41.4
Snacks				Snacks				Snacks		
Salmon and Spinach Salad	155	107.4		Pumpkin & Garlic Soup	234	81.9		Minty Tapenade	180	23.1
(Salmon, Lime, Spinach, Fat-free yogurt, Onion, Capers)				(Pumpkin, Onion, Garlic, Vegetable stock, Coconut cream, Almond butter, Ginger)				(Black olives, Mint, Avocado oil, Coconut cream)		
Lunch				Lunch				Lunch		
Lentil Medley	225	35.6		Veggie Pita Rolls	334	28.5		Roasted Kabocha with Wild Rice	250	64.2
(Lentils, Onion, Mushrooms, Potato, Toato, Cucumber, Spinach, Fresh mint, Honey, Fice vinegar, Olive oil)				(Whole-wheat pita breads, Lettuce, Cucumber, Tomato, Onion, Hummus, Bell pepper, Bell pepper, Olive oil, Lime juice)				(Kabocha squash, Wild Rice, Pumpkin seeds, Pomegranate seeds, Parsley, Lime juice, Honey, Lime zest, Olive oil, Chili powder, Pepper)		
Bread (Whole wheat bread)	226	5.2		Lunch subtotal	334	28.5		Bread (Whole wheat bread)	113	2.6
Lunch subtotal	451	40.7						Lunch subtotal	363	66.7
Snacks				Snacks				Snacks		
Italian Style Mushroom Mix	96	42.6		Sour Cream Green Beans	360	20.4		Honey Sage Carrots	217	1.8
(Mushroom, Onion, Tomato sause, Olive oil, Italian seasoning)				(Green Beans, Corn, Mushroom, Cream of mushroom soup, Low-fat sour cream, Almonds, Low-fat cheddar cheese)				(Carrots, Honey, Butter, Fresh sage)		
Dinner				Dinner				Dinner		
Shallot and Salmon Mix	369	143.8		Chicken with Tomatoes and Celery Stalk	265	335.4		Garlic Pork	344	187.6
(Salmon fillets, Shallot, Olive oil, Parsley)				(Chickedn breast, Celery, Tomato, Onion, Zucchini, Mushroom, Garlic, Olive oil)				(Pork chop, Onion, Potato, Olive oil, Sweet paprika, Garlic powder, Salt )		
Bread (Whole wheat bread)	226	5.2		Bread (Rye bread)	151	3.4		Bread (Whole wheat bread)	226	5.2
Dinner subtotal	595	148.9		Dinner subtotal	416	338.8		Dinner subtotal	570	192.8
Daily total	1713	350.9		Daily total	1600	493.9		Daily total	1630	325.9

## 4. Discussion

Gout and hyperuricemia are related to lifestyle habits, as the Japanese Guidelines for the Treatment of Hyperuricemia and Gout recommends lifestyle advice. Lifestyle advice includes dietary therapy, alcohol restriction, and exercise recommendations. Within this dietary therapy it recommends appropriate energy intake, avoidance of excessive intake of purine and fructose, and appropriate drinking of water according to renal function are recommended [5]. However, there is little information available regarding the daily intake of purine, making it difficult to visualize.

Thus, in this study, we summarized the purine content of commonly used food ingredients (Table 1), created an illustration based on the Japanese Food Guide Spinning Top (Figure 4), and calculated the amount of purine in well-balanced diets, including the Mediterranean diet and the DASH diet (Table 2, Table 3, Table 4 and Table 5).

The amount of purine across these recipes and in the image diagram in Figure 4 ranged from 308.5 to 493.9 mg/day. A recipe that used a lot of chicken had more than 400 mg. The amount of purine will vary depending on the ingredients used even in a well-balanced diet. By consuming 80–100 g of meat or fish per meal that contains high purine and protein, the amount of purine in well-balanced diet is thought to be around 400 mg/day.

The energy intake of these recipes ranged from 1510 to 2287 kcal, showing considerable variation. When comparing purines and energy across these recipes, the correlation is not clear. The energy of food depends on its fat and carbohydrate content, while purines are correlated with cell number.

Most gouty patients are men, and the recommended daily energy intake based on the average age, height, and weight of gouty patients is about 2200 kcal. If the energy intake in Japanese diet (Table 2) is increased to 2200 kcal and the amount of purine is also increased, the amount of purine in Japanese diet will be 365.9–419.9 mg. Similarly, in the My Plate Plan diet (Table 3), the amount of purine in that will be 361.6–370.7 mg. In the case of the Mediterranean diet (Table 4) and the DASH diet (Table 5), the amount of purine in them will be 360.5–509.0 mg and 439.9–679.1 mg, respectively. The Mediterranean diet and the DASH diet have a slightly higher purine content (converted a daily calorie intake to 2200 kcal), but these recipes contain many low-calorie ingredients. Since energy and purine are not directly correlated, it may not be meaningful to convert them to 2200 kcal and compare them. In any case, a balanced diet is believed to provide adequate energy and purine amount by following the ingredients and frequency recommended in the guidelines.

Regarding the purine content of commonly used ingredients (Table 1), as there are a few tables reporting purine content in foodstuffs, it is hoped that this table will be useful on providing dietary advice not only for patients with gout and hyperuricemia but also for the general public. There are still several ingredients that are lacking, so it is necessary to increase the number of foods and continue measuring them.

Figure 4 represents “A well-balanced diet based on the Japanese Food Guide Spinning Top”. This illustration is designed to allow users to present a diagram for intuitive understanding, and we hope that many people will find it useful.

We selected four dietary guidelines for determining purine content: the Japanese Food Guide diet, the My Plate Plan diet, the Mediterranean diet, and the DASH diet [23,24,25,26,27,28,29,30]. The reason is that these dietary guidelines are promoted as healthy diets by government agencies involved in national health of countries with many patients with gout and hyperuricemia, and the Mediterranean diet and the DASH diet have been reported to be effective for patients with gout and hyperuricemia [3,14,15,16,17,18]. Although it would have been better to refer to other dietary guidelines, we focused on these because well-balanced diets are well thought out and are considered to not vary significantly with each other.

This study has two features. The first is that we provide a list of the purine content of commonly used foodstuffs. We have been continuously measuring purine content, and a table compiling the data are useful (Table 1). The second is that we calculate the daily amount of purine contained in a generally recommended balanced diet. The balanced diet described in the Dietary Guidelines contains approximately 400 mg/day of purine. It is known that a high purine diet increases serum uric acid levels and increases the recurrence rate of gout attacks. A well-balanced diet is recommended not only to the general public but also to patients with gout and hyperuricemia.

There are two limitations to this study. The first is that there is still a lack of data on the purine content of foodstuffs. Further measurements are planned. The second is that the dietary guidelines used in this study were limited to four guidelines, not those from around the world. The reason for this was mentioned above, but another reason is that it was difficult to obtain foodstuffs from overseas, making it difficult to measure the purine content in them.

In the future, we plan to obtain various foodstuffs from around the world via the Internet and other means, measure the purine content in them, and use the results to provide dietary advice to patients with gout and hyperuricemia.

## 5. Conclusions

After creating recipes based on the dietary guidelines and calculating the amount of purine, the purine amount in the Japanese diet, the American My Plate Plan, the Mediterranean diet, and the DASH diet were found to be 308.5–493.9 mg/day. The diets examined in this study, the daily amount of purine did not exceed 400 mg/day, except for a recipe that included 200 g of chicken. A well-balanced diet following the recommendation of the dietary guidelines is considered to contain an adequate amount of purine, around 400 mg/day. Because a high purine diet can lead to the recurrence of gout, advising to eat a balanced diet should be useful lifestyle guidance for patients with gout and hyperuricemia.

## Figures and Tables

**Table 1:** Purine content of commonly used foodstuffs.

(mg/100g)			(mg/100g)			(mg/100g)			(mg/100g)	
Grains	Purine		Vegetables(colored)	Purine		Meat	Purine		Fresh Fish	Purine
Barley	44.3		Balsam pear (Goya)	9.9		Pork			Anchovy	272.8
Bread	4.4		Broccoli	61.9		Shoulder	81.4		Bonito	211.4
Buckwheat	7.7		Carrot	2.2		Ribs	75.8		Codfish	98
Dumpling Wrappers	13.4		Cilantro	39.5		Tenderloin	119.7		Flounder (Hirame)	133.4
Flour (bread flour)	25.8		Green beans	7.4		Sirloin	90.9		Halibut	113
Flour (cake flour)	15.7		Green pepper	2.4		Liver	284.8		Japanese Salmon	119.3
Oatmeal	35		Komatsuna	10.6					Japanese seabass	119.5
Rice (polished)	25.9		Mitsuba	11.6		Beef				
Rice (unpolished)	37.4		Parsley	288.9		Shoulder ribs	77.4		Pacific saury	154.9
Rice (with the bud)	34.5		Perilla leaves (shiso)	41.4		Shoulder sirloin	90.2		Rainbow trout	180.9
Spaghetti	6.8		Radish (leaf)	33.6		Shin	106.4		Red seabream	128.9
Udon	12.1		Red Paprika	1		Tenderloin	98.4		Salmon	176.5
			Spinach	51.4		Thigh	110.8		Tuna	157.4
Beans	Purine					Liver	219.8			
Azuki bean (dried)	77.6		Vegetables (other)	Purine		Chicken			Seafood	Purine
Black bean (dried)	67.1		Asparagus	32.8		Wing	137.5		Pacific flying squid	186.8
Broad bean	35.5		Bamboo shoot	47.1		White meat	153.9		Spear squid	160.5
Chickpeas	26.1		Bean sprouts	35		Leg	122.9		Octopus	137.3
Green peas	21.8		Cabbage	3.2		Breast	141.2		Shiba shrimp	144.2
Green peas (canned)	18.8		Cauliflower	57.2		Liver	312.2		Tiger Prawn	192.3
Green Soybean	47.9		Cherry Tomato	3.1		Mutton			Snow crab	136.4
Red peas	25.4		Chinese cabbage	7		Mutton	96.2		Clam	145.5
Soybean (dried)	172.5		Corn	11.7		Rum	93.5		Oyster	184.5
			Cucumber	9.4		Duck	163.9		Mussels	292.5
Soybean products	Purine		Eggplant	50.7		Horse	140.8		Scallop	104.9
Fermented (Natto)	56.6-113.9		Garlic	17		Venison	140.4		Kamaboko	26.4
Soymilk	19.3-22.0		Ginger	2.3		Wild boar	91.7		Katsuobushi (flake)	493.3
Tofu	20.0-31.1		Japanese leek (negi)	41.4		Rabbit	130.7			
			Lettuce	4.6		Boneless ham	74.2		Seasoning	Purine
Nuts/Seeds	Purine		Mizuna	20.2		Vienna sausage	45.5		Barbecue sauce	14.9
Almond	31.4		Onion	2.3		Bacon	74.8		Chicken soup stock	508.9
Chia Seed	58.6		Radish (root)	1.7					Consomme (powder)	179.8
Peanut	49.1		Tomato	6.5		Fruit	Purine		Honey	0.9
Sesame	36.3		Zucchini	13.1		Avocado	18.4		Ketchup	10.5
Walnut	19.6					Banana	3		Mayonnaise	0.6
			Potatoes	Purine		Kiwi	1.8		Mirin	1.2
Eggs	Purine		Pumpkin (Kabocha)	56.6		Mandarin Orange	1.7		Miso (red miso)	63.5
Chicken egg	0		Potato	6.5		Strawberry	2.1		Miso (white miso)	48.8
			Sweet potato	17					Shishito pepper	5.1
Dairy products	Purine					Beverages	Purine		Soy sauce	45.2
Butter	0		Mushrooms	Purine		Fruit juice	1.1		Tomato paste	10.9
Cheese	6		Bunashimeji	20.8		Tomato juice	6.2		Umami broth (Bonito)	684.8
Fresh cream	0.3		Eringi	13.4		Vegetable juice	13.7		Wasabi	0.7
Fresh cream (veg)	0.1		Mushroom	49.5		Japanese sake	1.5			
			Shiitake (raw)	20.8		Red Wine	1.6			
Grated cheese	12.9					White Wine	1.6			
Margarine	0		Dried seaweeds	Purine						
Milk	0.16		Hijiki	132.8		Others	Purine			
Milk (low fat)	0.15		Kombu	46.4		Chocolate	8.1			
Yogurt	5.2		Nori	591.7		Konjac	0.3			
			Wakame	262.4		Vermicelli	0.6			

## Data Availability

The original contributions presented in this study are included in the article. Further inquiries can be directed to the corresponding author.
